# Pauses, silences, and cognitive control: psycholinguistic markers of speech planning in interpreter training

**DOI:** 10.3389/fpsyg.2026.1775771

**Published:** 2026-05-19

**Authors:** Hua Cai, Ayob Leelany

**Affiliations:** School of Humanities, Universiti Sains Malaysia, Gelugor, Pulau Pinang, Malaysia

**Keywords:** cognitive control, dual mechanisms framework, paralinguistic cognition, metacognitive training, interpreting, randomized controlled trial

## Abstract

Cognitive control stands at the heart of goal-directed behavior. Braver’s Dual Mechanisms of Control framework distinguishes between proactive and reactive control, yet its applicability in complex speech production and whether cognitive control strategies can be trained remain underexplored. Consecutive interpreting, as an extreme cognitive control task, provides a unique window for examining these questions. This study investigated whether paralinguistic cognitive training could facilitate a shift from reactive to proactive control in interpreters, while testing the validity of paralinguistic features as indicators of cognitive control strategies. A randomized controlled trial was conducted with 60 undergraduate interpreting students randomly assigned to an experimental group and a control group (30 participants each). The experimental group received paralinguistic cognitive training beyond conventional training, whereas the control group underwent standard instruction only. Data were collected at pre-test (Week 1) and post-test (Week 13), with paralinguistic features annotated using ELAN software. Mixed-design analysis of variance examined group by time interaction effects. The experimental group demonstrated significant improvements: pause frequency dropped by 63%, lengthening frequency fell to zero in the English-to-Chinese direction, and self-repair frequency declined by 28%. The control group exhibited intensified reactive control characteristics—self-repair frequency rose by 35% and pause duration extended by 25%. Interaction effects revealed that self-repair frequency showed the most pronounced between-group divergence with large effect sizes, displaying a classic scissor-pattern differentiation. This study provides empirical support for applying Braver’s Dual Mechanisms of Cognitive Control framework to speech production, confirming that cognitive control strategies can be trained to shift from reactive toward proactive modes. These findings extend the framework’s application boundary while offering new evidence for intervention research on enhancing cognitive control through metacognitive training.

## 1 Introduction

Cognitive control constitutes a core capacity enabling humans to perform complex cognitive tasks—it refers to the psychological processes through which individuals flexibly regulate thought and behavior in line with internal goals. Braver (2012) Dual Mechanisms of Cognitive Control framework distinguishes two complementary modes: proactive control, which optimizes processing readiness by activating and maintaining goal-relevant information in advance, and reactive control, which temporarily mobilizes cognitive resources only after detecting conflict or error. This theoretical framework has been extensively validated through classic experimental paradigms such as task switching, the Stroop task, and AX-CPT, yet its applicability in continuous, dynamic speech production contexts awaits further investigation. Consecutive interpreting, a speech production task placing extreme demands on cognitive control, provides an ideal research context for testing the ecological validity of the dual mechanisms framework.

From a psycholinguistic perspective on speech production, Levelt (1989) classic model posits that speech output involves three successive processing stages—conceptualization, formulation, and articulation—each requiring the mobilization of corresponding cognitive resources alongside real-time monitoring and adjustment. Kormos (2006) extended this model to bilingual contexts, emphasizing that fluent speech production requires speakers to engage in effective prospective planning during the conceptualization stage to reduce correction needs during output. Bosker et al. (2013) investigated the relative contributions of pauses, speech rate, and repairs to fluency perception, finding pauses to be the strongest predictor of fluency judgments. Within this theoretical framework, paralinguistic features such as pauses, silences, and self-repairs serve as overt markers of cognitive difficulty during speech processing. Betz et al. (2023) directly confirmed the causal relationship between cognitive load and speech hesitation through experimental research. Fraundorf and Watson (2011) further demonstrated that filled pauses not only reflect speakers’ cognitive states but also affect listeners’ memory encoding processes; Arnold et al. (2007) used eye-tracking to discover that listeners exploit speakers’ disfluency features to make online inferences about referent processing difficulty, revealing the bidirectional communicative function of paralinguistic features. Integrating Levelt-Kormos’s speech production model with Braver’s dual mechanisms control framework suggests a new theoretical perspective: high frequencies of pauses, silences, and self-repairs may reflect speakers’ over-reliance on reactive control, while targeted training to enhance proactive control capability could fundamentally improve speech production fluency.

Working memory, as the core infrastructure of cognitive control, performs critical buffering and integration functions during speech production. Baddeley (2000) working memory model emphasized the central executive system’s crucial role in coordinating attentional resources and monitoring cognitive processing. Oberauer (2019) systematic analysis of the relationship between working memory and attention indicated that individual differences in working memory capacity largely reflect variations in attentional control ability—a view supported by Engle (2018) theoretical integration of working memory and executive attention. Etzel et al. (2022) released a dual mechanisms cognitive control dataset providing standardized methodological reference for examining relationships between working memory and cognitive control strategies. Mellinger and Hanson (2021) meta-analysis confirmed robust positive correlations between working memory capacity and complex verbal task performance; Ghiselli (2022) further examined the predictive validity of different working memory tasks through meta-analysis. However, whether working memory capacity can be enhanced through training and whether cognitive control strategies can shift from reactive to proactive modes remain questions of significant theoretical importance for understanding the boundaries and mechanisms of cognitive plasticity.

Regarding the specific working memory components most relevant to interpreting performance, Mellinger and Hanson (2021) meta-analysis identified phonological working memory and central executive updating capacity as the most consistently predictive WM components across consecutive and simultaneous interpreting tasks, with effect sizes indicating medium-to-large practical significance. Ghiselli (2022) meta-analysis further demonstrated that tasks measuring executive attention and controlled processing show greater predictive validity for interpreting performance than purely storage-based WM measures, underscoring the central role of attentional control within working memory for complex language production tasks. Dong et al. (2018) provided direct longitudinal evidence by assessing phonological loop capacity (digit span, word span) and central executive updating (n-back, reading span) in interpreting trainees, finding training-induced gains across both storage and processing components—suggesting that the WM subsystems most predictive of interpreting performance are also responsive to training.

Paralinguistic features as behavioral markers of cognitive processing states have garnered fairly rich empirical support. Plevoets and Defrancq (2018) large-scale corpus analysis found that information load indicators significantly predicted the occurrence frequency of disfluency markers in speech production. Engelhardt et al. (2019) employed latent variable analysis to examine individual differences in disfluent production, discovering that working memory ability and verbal intelligence explained significant portions of disfluency variance. Oliveira et al. (2023) demonstrated that processing fluency impairs cognitive control adjustment—when individuals experience processing fluency, they become more prone to relying on automatic responses. Han and An (2021) found that adopting different measurement thresholds significantly affects disfluency indicator measurement outcomes. Bartłomiejczyk and Gumul (2024) further noted that different types of disfluency phenomena may reflect different levels of cognitive processing. These studies collectively suggest that paralinguistic features can serve as effective behavioral indicators of cognitive control strategies, providing operationalizable measurement tools for testing the dual mechanisms framework.

In the domain of professional interpreting specifically, Song and Cheung (2019) demonstrated through corpus-based analysis of United Nations General Assembly data that disfluency patterns—including filled pauses, repairs, and output dispersion—vary systematically between relay and non-relay simultaneous interpreting, indicating that different cognitive demands elicit distinct control responses. Fan et al. (2025) further reported that consecutive and simultaneous interpreting impose differential cognitive saturation on learners, as reflected in variations in lexical and syntactic complexity assessed through entropy-based measures. Together, these findings validate paralinguistic and linguistic features as reliable indicators of cognitive control processes in professional language production, providing an empirical foundation for the present investigation of whether targeted training can modulate such features to facilitate proactive control.

The plasticity of cognitive control ability represents one of the core issues in cognitive psychology, directly concerning the theoretical foundations of cognitive training and intervention research. Rodas et al. (2024) meta-analysis systematically examined the effects and biases of working memory training, finding significant task specificity in training effects with relatively limited far transfer. In the domain of speech production, Hervais-Adelman and Babcock (2021) explored the neural mechanisms of cognitive control in extreme language control contexts from a neurobiological perspective. Dong and Li (2021) proposed an attentional control model integrating language control and processing control, emphasizing dynamic cognitive resource allocation. Nour et al. (2021b) systematic review examined the impact of intensive cognitive training on executive function, finding that specialized training can significantly shape cognitive control ability; another study by the same team (Nour et al., 2021a) further confirmed training effects on working memory adaptive control. These studies collectively suggest that cognitive control is not a fixed trait but rather a cognitive skill that can be enhanced through specialized training.

Metacognition, as “cognition about cognition,” involves individuals’ monitoring and regulation of their own cognitive processes and serves as a key mechanism for achieving flexible switching of cognitive control strategies. Flavell (1979) defined metacognition as knowledge and cognition concerning cognitive phenomena, emphasizing the critical role of metacognitive monitoring in learning and problem-solving. Fleming and Dolan (2012) systematically articulated the neural foundations of metacognitive ability from a neuroscience perspective, revealing the prefrontal cortex’s critical role in metacognitive monitoring. Boldt and Gilbert (2022) neuroimaging research uncovered partially overlapping yet separable neural bases for metacognitive monitoring and metacognitive control, providing neural-level evidence for understanding the dual functions of metacognition. In speech production contexts, speakers’ metacognitive awareness and regulatory capacity regarding their own speech output may be an important factor influencing paralinguistic features—individuals with high metacognitive awareness can detect potential production difficulties earlier, thereby mobilizing cognitive resources in advance for preparation, reflecting characteristics of proactive control. Chmiel et al. (2020) found through eye-tracking that professional speech task performers possess unique advantages in cognitive monitoring. Bakti and Bóna (2016) research discovered that under high cognitive load conditions, speakers tend to extend pause duration to gain additional processing time, suggesting that paralinguistic features are not merely passive reflections of cognitive processing states but may also serve as strategic tools individuals use to actively regulate speech production.

Consecutive interpreting, as an extreme cognitive control task, provides an ideal natural experimental context for testing the above theoretical questions. This task requires individuals to complete real-time cross-linguistic information conversion under strict time pressure, with cognitive resources typically operating at critical saturation levels (Gile, 2009), such that any differences in cognitive control strategies become amplified and overtly manifest at the behavioral level. Dong et al. (2018) longitudinal study found that training can promote enhancement of specific working memory components. Lin et al. (2018) demonstrated that working memory can explain 50–51% of speech disfluency variance. However, the above research has focused primarily on changes in cognitive ability itself, with relatively insufficient attention to the trainability of paralinguistic features as overt indicators of cognitive control strategies, particularly lacking attempts to integrate the dual mechanisms cognitive control framework with paralinguistic phenomena. Consecutive interpreting was specifically selected over simultaneous interpreting as the experimental mode for three theoretically motivated reasons. First, CI temporally separates the comprehension-and-note-taking phase from the production phase, creating a discrete speech planning window that renders cognitive control strategies more behaviorally visible and measurable. Second, unlike simultaneous interpreting—where listening and speaking proceed concurrently with perpetually divided attentional resources—CI permits clearer attribution of paralinguistic features to the speech planning stage rather than to input-processing interference. Third, CI is typically introduced in the early stages of interpreter training curricula, making it methodologically appropriate for examining training-induced cognitive shifts in novice learners.

**Figure 1:**
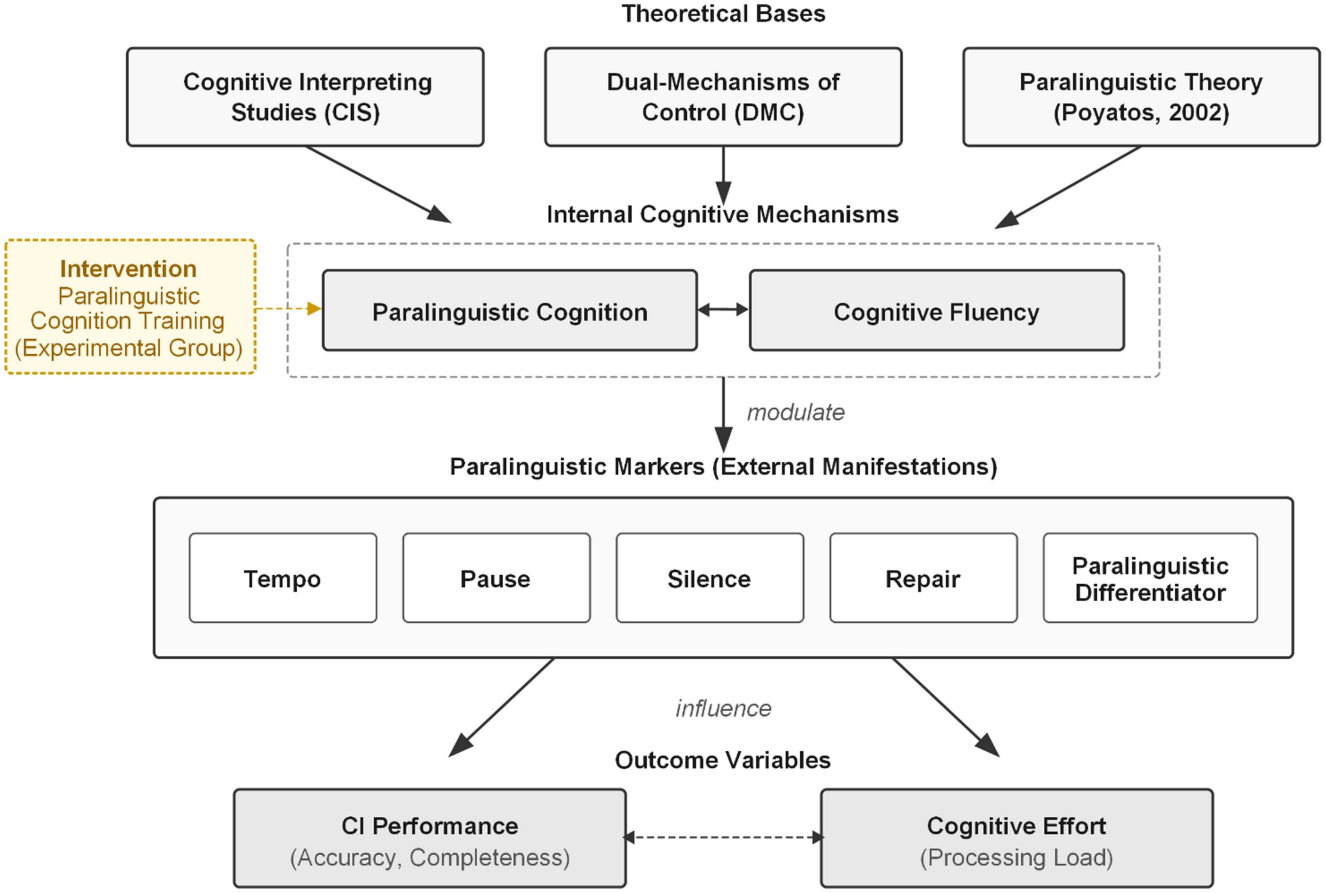
Theoretical framework of paralinguistic markers in consecutive interpreting. Solid lines indicate direct relationships; dashed lines indicate interactive or moderating relationships. The intervention (paralinguistic cognition training) is applied only to the experimental group.

Addressing the above theoretical gaps, this study integrates Braver (2012) Dual Mechanisms of Cognitive Control framework, the Levelt-Kormos speech production model, and Flavell (1979) metacognition theory to propose that paralinguistic cognitive training may enhance individuals’ metacognitive awareness and active regulatory capacity regarding paralinguistic features, thereby facilitating a strategic shift from reactive to proactive control and consequently improving speech planning efficiency and production fluency. This study selected consecutive interpreting tasks as the experimental context for testing these theoretical hypotheses and constructed a theoretical analytical framework for paralinguistic markers (Figure 1). This framework treats paralinguistic cognition and cognitive fluency as interacting internal cognitive mechanisms that jointly modulate overt paralinguistic markers such as pauses, silences, and self-repairs.

Based on the above theoretical framework, this study proposes the following hypotheses: H1: As external markers of speech planning processes, the frequency and duration of pauses and silences can effectively reflect individuals’ cognitive control strategies; H2: Following systematic paralinguistic cognitive training, the experimental group’s paralinguistic features will show regular improvement, manifesting as significant decreases in pause, silence, and self-repair indicators, reflecting a strategic shift from reactive to proactive control; H3: The improvement effects of paralinguistic cognitive training will significantly exceed those of conventional training, manifesting as statistically significant group × time interaction effects.

The theoretical contribution of this study lies in extending Braver’s dual mechanisms cognitive control framework to the complex speech production domain, testing the framework’s applicability in high ecological validity contexts, and validating through a randomized controlled trial design the trainability of cognitive control strategy shifts from reactive to proactive. This research not only deepens theoretical understanding of cognitive control plasticity but also provides new empirical evidence and methodological reference for intervention research aimed at enhancing cognitive control ability through metacognitive training.

## 2 Materials and methods

### 2.1 Participants

This study employed purposive sampling to recruit third-year undergraduate students from the School of Foreign Languages at a Chinese university who were enrolled in consecutive interpreting courses (English-Chinese/Chinese-English bidirectional) as research participants. Inclusion criteria included: passing an entrance examination to ensure adequate bilingual proficiency, with no prior systematic interpreting training. The final valid sample comprised 60 participants randomly assigned to an experimental group (*n* = 30) and a control group (*n* = 30). The two groups showed no significant differences in baseline characteristics including age, gender distribution, English proficiency test scores, and prior interpreting course credits (Table 1), validating the effectiveness of random assignment.

**Table 1:** Participant characteristics.

Characteristic		Experimental group (*n* = 30)	Control group (*n* = 30)	Statistics
Gender, *n* (%)	Female	19 (63.3)	15 (50.0)	X^2^ = 1.11, *p* = 0.29
	Male	11 (36.7)	15 (50.0)	
Age, years	*M* (*SD*)	21.2 (0.8)	21.1 (0.7)	*t* = 0.52, *p* = 0.60
	Range	20–22	20–22	
Native language	Chinese	30 (100)	30 (100)	/
Second language	English	30 (100)	30 (100)	/
Education level	Undergraduate Year 3	30 (100)	30 (100)	/
Prior interpreting training	None	30 (100)	30 (100)	/

M = mean; SD = standard deviation. All participants were native Chinese speakers with English as their second language, enrolled in consecutive interpreting courses (E-C and C-E).

Sample size was determined through *a priori* statistical power analysis. This study used G*Power 3.1 software to estimate sample size for a 2 (group: experimental vs. control) × 2 (time: pre-test vs. post-test) mixed-design repeated measures ANOVA in a randomized controlled trial. Parameter settings followed effect size standards from cognitive control training research (Braver et al., 2021): effect size *f* = 0.25 (medium effect), *α* = 0.05, statistical power 1 − *β* = 0.80, between-group correlation r = 0.50. Calculations indicated a minimum required sample size of 52 participants; considering potential attrition, this study ultimately recruited 60 participants to ensure statistical power. This research was approved by the Universiti Sains Malaysia Ethics Committee (Approval No.: USM/JEPeM/PP/25040333). All research participants signed written informed consent before participating, and were informed they could withdraw unconditionally at any stage.

### 2.2 Experimental materials

Experimental material design followed principles combining ecological validity with experimental control. This study selected consecutive interpreting tasks as the experimental context for testing cognitive control strategies, based on the following theoretical considerations: consecutive interpreting requires individuals to complete real-time cross-linguistic information conversion under strict time pressure, with cognitive resources typically operating at critical saturation levels (Gile, 2009), such that any differences in cognitive control strategies become amplified and overtly manifest at the behavioral level, thereby providing a highly sensitive measurement window for testing the dual mechanisms cognitive control framework.

The experiment employed a bidirectional consecutive interpreting task design, including English-to-Chinese (E-C) and Chinese-to-English (C-E) directions. All materials focused on the theme of artificial intelligence to control the influence of topic variables on cognitive processing. English materials were selected from Elon Musk’s speech at the 2023 World Artificial Intelligence Conference; Chinese materials were selected from a speech by Huawei’s rotating chairman Hu Houkun. Material difficulty was matched through indicators such as lexical density, syntactic complexity, and information density to ensure comparability across directions. Audio materials were recorded by native speakers under standardized conditions, with each task lasting no more than 1.5 min and 2-min rest intervals between tasks to reduce fatigue effects.

### 2.3 Research design and procedures

**Figure 2:**
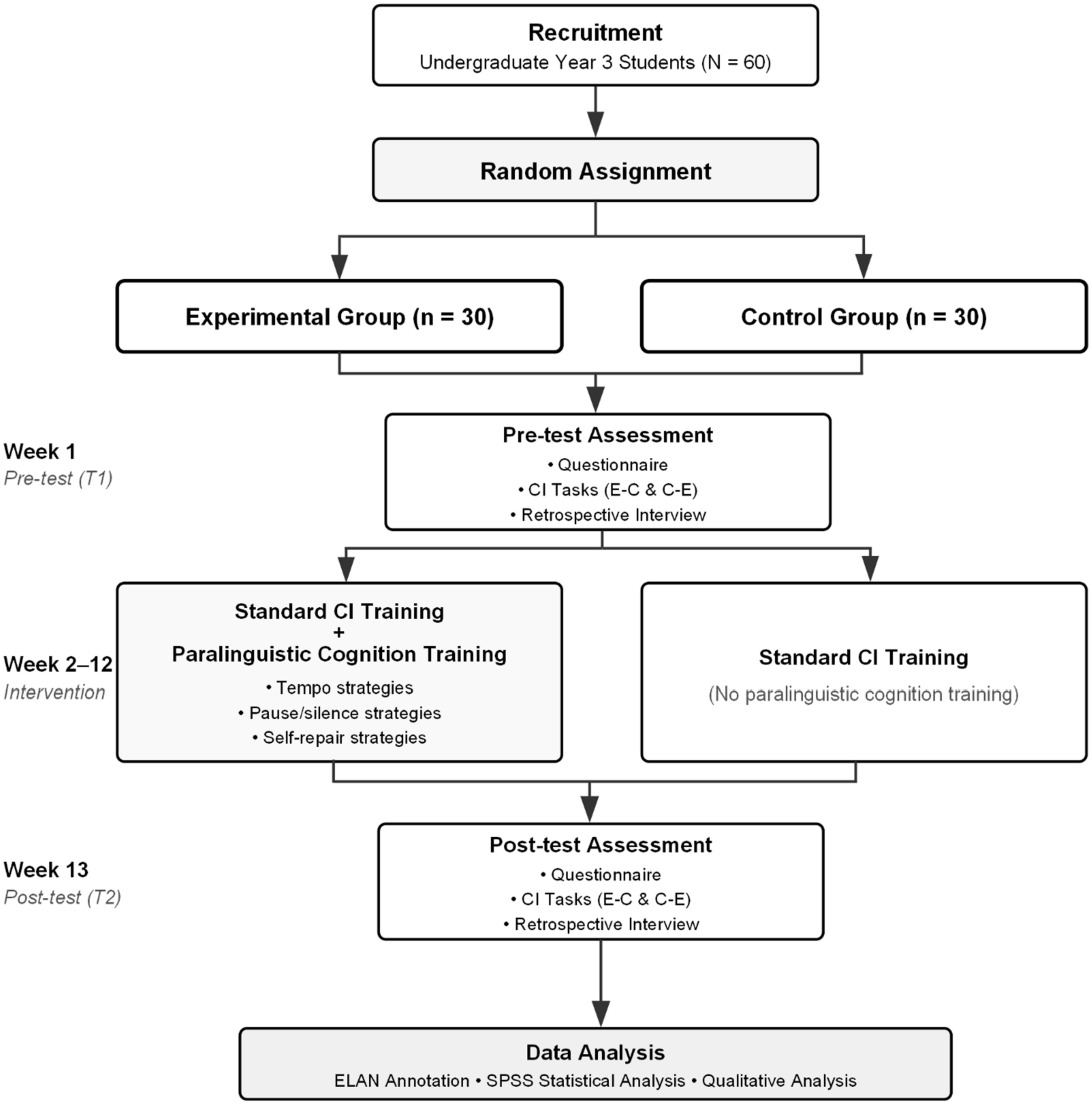
Research design procedure. CI = consecutive interpreting; E-C = English to Chinese; C-E = Chinese to English.

This study employed a 2 × 2 mixed-design randomized controlled trial, with group (experimental/control) as the between-subjects variable and time (T1/T2) as the within-subjects variable (Figure 2). This design enables simultaneous examination of both the main effect of paralinguistic cognitive training and its interaction with time, thereby addressing the core question of “whether the experimental group’s cognitive control strategy shift is significantly superior to the control group.” Pre-test (T1) was conducted in Week 1, post-test (T2) in Week 13, with the intervention phase lasting 12 weeks. The experimental group received a dual-track curriculum of conventional training plus paralinguistic cognitive training during the intervention phase, while the control group received conventional training only. Pre-test and post-test employed the same material design framework but used different specific texts to avoid practice effects contaminating results.

The experimental procedure followed a standardized pre-test–intervention–post-test paradigm. During the pre-test phase (T1), all participants completed baseline tasks in a standardized laboratory environment. The experimental environment controlled for room temperature (22–24 °C) and ambient noise (<40 decibels), with participants seated separately to avoid mutual interference. Each participant completed bidirectional consecutive interpreting tasks (multiple passages for both English-to-Chinese and Chinese-to-English), with task presentation order randomized to control for order effects.

The paralinguistic cognitive training during the intervention phase was designed by the research team based on Flavell (1979) metacognition theory, Braver (2012) dual mechanisms control framework, and Kormos (2006) speech production model. Spanning 12 weeks, it aimed to enhance individuals’ metacognitive awareness and active regulatory capacity regarding paralinguistic features while facilitating their strategic shift from reactive to proactive control. The training covered four core modules. The speech rate and prosody control module trained individuals to convey different semantic functions and emotional tones through regulating speech rate and pitch. The pauses and silences module helped individuals distinguish between hesitation pauses and planning pauses, training them to perceive functional differences in pauses between source and target languages, and learning to use pauses as information organization tools. The self-repair module, based on Tang (2020) classification framework, trained individuals to identify and appropriately apply three types of repair behaviors—input-driven, output-driven, and interpreter-driven—to reduce unnecessary post-hoc repairs. The paralinguistic differentiators module trained individuals to perceive and produce functional sounds such as laughter, coughing, and throat clearing. Training adopted a blended learning format, with learning materials (theoretical explanations, annotated examples, practice audio) and practice tasks released weekly through an online platform, and the research team providing individualized feedback on submitted exercises. The control group received only conventional interpreting training without paralinguistic cognitive training content. Drawing on established working memory frameworks, we propose the following theoretically motivated connections between training modules and WM components: Module 1 (speech rate and prosody control) is hypothesized to engage phonological loop rehearsal processes and attentional monitoring of articulatory output (Baddeley, 2000; Kormos, 2006); Module 2 (pauses and silences) is hypothesized to tap central executive functions, particularly proactive attentional resource allocation and self-monitoring capacity (Oberauer, 2019); Module 3 (self-repair) is hypothesized to engage episodic buffer integration, online error detection, and metacognitive self-correction routines (Roebers, 2017; Engelhardt et al., 2019); Module 4 (paralinguistic differentiators) is hypothesized to draw on attentional filtering and controlled processing of non-verbal communicative signals (Engle, 2018). It should be noted that the cited works provide theoretical characterizations of these WM components and their roles in cognitive processing and speech production; the specific mappings between our training tasks and these components are proposed by the present authors on the basis of theoretical reasoning rather than derived from studies that have directly tested these links. Representative training materials, including sample task stimuli and annotated response examples for each module, are provided in Supplementary File S1. Each module spanned approximately 3 weeks.

The post-test phase (T2) procedures, testing environment, and data collection protocols remained strictly consistent with pre-test to ensure measurement comparability. Both groups completed tasks matched in difficulty to pre-test but with different content.

### 2.4 Measurement indicators

The dependent variables in this study included two major categories: task performance and paralinguistic performance, with operational definitions for each variable detailed in Table 2. Task performance was evaluated through two indicators: percentage of correctly translated vocabulary and percentage of untranslated meaning units, referencing Gile (2009) quality assessment framework and the competence scale developed by Wang et al. (2020) to ensure indicator validity and reliability. Evaluation was completed independently by two experienced raters blind to participants’ group assignments to avoid rating bias. Inter-rater reliability was examined using intraclass correlation coefficient (ICC), with results showing ICC = 0.91, indicating excellent rating consistency.

**Table 2:** Operational definitions of variables.

Variable type	Variable	Operational definition
Independent variables	Group	Experimental group (standard CI training + paralinguistic cognition training) vs. Control group (standard CI training only)
	Time	T1 (Week 1, pre-test) vs. T2 (Week 13, post-test)
Dependent variables	Interpreting performance	
	Accuracy	Percentage of words/characters correctly interpreted, calculated as (correctly interpreted words ÷ total source words) × 100%
	Completeness	Percentage of meaning groups left uninterpreted, calculated as (omitted meaning groups ÷ total meaning groups) × 100%
	Paralinguistic performance	
	Drawling	Mean length (seconds) and frequency (per minute) of vowel or consonant prolongations in target speech
	Pauses (filled)	Vocalized hesitations (e.g., “uh,” “um”); threshold ≥ 0.2 s; short pause: 0.2–1.999 s; long pause: ≥ 2.0 s; measured by mean length (seconds) and frequency (per minute)
	Silences (unfilled)	Silent intervals in speech flow; threshold ≥ 0.2 s; short silence: 0.2–1.999 s; long silence: ≥ 2.0 s; measured by mean length (seconds) and frequency (per minute)
	Self-repairs	Self-initiated corrections in target speech; measured by frequency (per minute) and distribution across three types
	Input-generated	Repairs to resolve inconsistencies between interpreter’s output and source speech content
	Output-generated	Repairs to increase pragmatic appropriateness or accuracy of target speech expression
	Interpreter-generated	Repairs (e.g., repetitions, restarts) to gain processing time during cognitive load
	Paralinguistic differentiators	Frequency of non-speech sounds (e.g., coughing, throat clearing, laughing) occurring during target speech production

Paralinguistic performance measurement covered five core indicator categories: lengthening, pauses, silences, self-repairs, and paralinguistic differentiators. These indicators are viewed as behavioral markers of cognitive control strategies: high frequencies of pauses, silences, and self-repairs may reflect reliance on reactive control, while decreases in these indicators may indicate a strategic shift toward proactive control. Lengthening indicators were measured through average duration and frequency per minute. The threshold for pauses and silences was set at 0.2 s, a threshold selection that comprehensively considered the modal characteristics of speech production and methodological recommendations from previous research (Plevoets and Defrancq, 2018; Han and An, 2021). Durations between 0.2 and 1.999 s were annotated as short pauses or short silences; durations of 2 s or longer were annotated as long pauses or long silences. Self-repair behavior measurement adopted Tang (2020) classification framework, with measurement indicators including total repair frequency per minute and distribution percentages across input-driven, output-driven, and interpreter-driven repair types. Paralinguistic differentiators—non-speech sounds including laughter, coughing, and throat clearing (Trager, 1958)—were measured as total frequency per minute, capturing involuntary physiological sounds as well as intentional communicative signals produced during interpreting output.

All paralinguistic features were annotated using ELAN 6.1 software. Annotation procedures followed standardized protocols: identifying onset and offset boundaries of paralinguistic features based on waveforms and spectrograms in segmentation mode, then conducting precise annotation and classification through repeated audio listening in transcription mode. To ensure annotation reliability, two systematically trained annotators independently completed all data annotation, with annotators blind to participants’ group assignments to avoid annotation bias. Subsequently, inter-rater consistency was examined on 20% of the data, with Cohen’s *κ* coefficient of 0.87, indicating excellent annotation consistency.

It should be noted that the primary quantitative analyses reported in this study are based on overall pause and silence frequency and duration, without a formal annotation layer separating planning pauses from hesitation pauses. However, to support the theoretical interpretation of pause-related findings within the dual mechanisms framework (see Discussion), we adopted a set of interpretive criteria derived from established disfluency research. Specifically, pauses occurring at major syntactic boundaries—at clause or sentence junctures—and immediately preceding new propositional content units are theoretically indicative of planning function, reflecting prospective cognitive preparation (proactive control). In contrast, pauses occurring mid-phrase or mid-clause, or co-occurring with filled pauses (uh/um), false starts, or restarts, are theoretically indicative of hesitation function, reflecting reactive conflict resolution. These criteria, informed by syntactic position and prosodic context, were used as an analytical lens for interpreting the dissociated patterns of pause frequency and duration observed between groups, rather than as a separate coding scheme applied during annotation. Future studies could implement these criteria as a formal annotation tier to enable direct quantitative comparison of planning versus hesitation pause distributions.

### 2.5 Data analysis

Statistical analyses were completed using IBM SPSS Statistics 26 software. Prior to formal analysis, all continuous variables underwent outlier screening (using ±3 standard deviations as the cutoff criterion) and normality testing (Shapiro–Wilk test). For within-group comparisons between the two time points, normally distributed data used paired-samples *t*-tests while non-normally distributed data used Wilcoxon signed-rank tests; between-group comparisons correspondingly used independent-samples *t*-tests or Mann–Whitney *U* tests. Effect sizes were quantified using Cohen’s *d* (parametric tests) or *r* (non-parametric tests), with effect sizes classified according to Cohen (1988) criteria as small (*d* = 0.2, *r* = 0.10), medium (*d* = 0.5, *r* = 0.30), and large (*d* = 0.8, *r* = 0.50).

To test whether paralinguistic cognitive training effects significantly exceeded conventional training, this study employed 2 (group) × 2 (time) mixed-design repeated measures ANOVA to examine group × time interaction effects on key dependent variables. Significant interaction effects indicate essential differences in change trajectories between the two groups, thereby providing statistical evidence for intervention effects. Partial η^2^ was used to quantify interaction effect sizes, classified according to Richardson (2011) criteria as small (η^2^p = 0.01), medium (η^2^p = 0.06), and large (η^2^p = 0.14). All statistical tests used two-tailed tests with significance level set at *α* = 0.05. Given that this study employed a theory-driven hypothesis testing framework and focused on effect size magnitude rather than relying solely on *p*-values for judgment, no statistical correction for multiple comparisons (such as Bonferroni correction) was applied. This decision was based on the following considerations: the study’s primary inferences relied on interaction effect tests rather than separate post-hoc comparisons, and overly conservative corrections might increase Type II error risk, obscuring genuine training effects. Result interpretation comprehensively considered statistical significance, effect size magnitude, and theoretical consistency. The choice of 2 × 2 mixed-design ANOVA over linear mixed-effects models (LME) was based on the following considerations: (1) the design is fully balanced with equal group sizes (*n* = 30 per group) and complete data at both time points—conditions under which ANOVA and LME yield statistically equivalent fixed-effect estimates; (2) the simple two-level repeated-measures structure does not necessitate the random-slopes parameterization that LME is principally designed to accommodate; (3) partial η^2^ provides standardized effect size indices widely reported in the applied linguistics and cognitive psychology literatures, facilitating direct comparison with prior interpreting research (Dong et al., 2018; Lin et al., 2018; Nour et al., 2021a; Nour et al., 2021b).

## 3 Results

### 3.1 Baseline comparisons and descriptive statistics

Baseline between-group comparison results validated the effectiveness of random assignment (Table 3). The two groups showed no statistically significant differences on any task performance or paralinguistic feature indicators (all *p* > 0.05), with between-group effect sizes for all indicators at the small effect level (Cohen’s *d* < 0.4), indicating successful random assignment and providing a valid foundation for subsequent intervention effect comparisons. Both groups exhibited typical characteristics of learner populations: moderate levels on task accuracy and completeness indicators, relatively high paralinguistic feature frequencies—this baseline pattern provides sufficient room for improvement to detect training effects.

**Table 3:** Descriptive statistics at T1 and T2.

Variable	Experimental group (*n* = 30)		Control group (*n* = 30)	
	T1	T2	T1	T2
E-C direction				
Interpreting performance				
Accuracy (%), *Mdn*	27	49	34	54
Uninterpreted (%), *Mdn*	82	41	69	44
Paralinguistic performance				
Drawling frequency (per min), *Mdn*	1.54	0.00	1.34	0.73
Drawling duration (s), *Mdn*	0.41	0.00	0.45	0.46
Pause frequency (per min), *Mdn*	10.85	4.01	9.79	9.56
Pause duration (s), *Mdn*	0.61	0.58	0.71	0.85
Silence frequency (per min), *Mdn*	17.09	10.51	11.96	8.05
Silence duration (s), *Mdn*	0.97	0.75	0.99	0.82
Repairs (per min), *M* (*SD*)	3.79 (1.63)	2.72 (1.40)	3.06 (2.02)	4.14 (2.20)
Paralinguistic differentiators (n)	11	6	7	7
C-E direction				
Interpreting performance				
Accuracy (%), *Mdn*	53	73	59	72
Uninterpreted (%), *Mdn*	54	20	43	20
Paralinguistic performance				
Drawling frequency (per min), *Mdn*	4.53	1.24	2.17	1.10
Drawling duration (s), *Mdn*	0.83	0.79	0.85	0.80
Pause frequency (per min), *Mdn*	7.39	4.23	6.85	5.92
Pause duration (s), *Mdn*	0.65	0.70	0.71	0.89
Silence frequency (per min), *M* (*SD*)	19.17 (3.81)	14.95 (4.11)	14.14 (5.58)	11.36 (4.90)
Silence duration (s), *Mdn*	1.08	1.11	0.99	1.27
Repairs (per min), *M* (*SD*)	3.80 (1.52)	3.73 (1.48)	4.02 (1.79)	4.46 (1.95)
Paralinguistic differentiators (*n*)	12	8	11	8

E-C = English to Chinese; C-E = Chinese to English; Mdn = median; M = mean; SD = standard deviation. Medians are reported for non-normally distributed variables (Wilcoxon signed-rank tests); means and standard deviations are reported for normally distributed variables (paired *t*-tests). Paralinguistic differentiators represent the total number of non-speech sounds (e.g., coughing, throat clearing, laughing) occurring during target speech production.

Regarding baseline levels of paralinguistic features, the two groups likewise demonstrated statistical comparability. Between-group differences in core indicators such as pause frequency, lengthening frequency, and self-repair frequency did not reach statistical significance (all *p* > 0.05). For silence frequency, in the English-to-Chinese direction, the experimental group’s values exceeded the control group’s (17.09 times/min vs. 11.96 times/min, 43% numerical difference), but this difference did not reach statistical significance (*p* > 0.05). Although random assignment statistically guaranteed baseline equivalence, this numerical difference suggests the need for caution when interpreting subsequent results regarding the potential influence of baseline imbalance. Random assignment ensured internal validity, and between-group differences emerging after training can be attributed to intervention effects rather than selection bias.

By T2, the two groups exhibited significantly divergent developmental trajectories. The experimental group showed consistent declining trends across core paralinguistic indicators including pause frequency, lengthening frequency, and silence frequency, with self-repair frequency significantly declining in the English-to-Chinese direction. Most indicators decreased by over 40%, and lengthening frequency in the English-to-Chinese direction dropped from baseline to zero. The control group’s change pattern presented more complex characteristics: some indicators remained stable (such as pause frequency), while others showed change directions opposite to the experimental group (such as extended pause duration and increased self-repair frequency). The experimental group’s improvement magnitude exceeded the control group’s on the vast majority of indicators; even for indicators where both groups showed declining trends (such as silence frequency), the experimental group’s effect sizes exceeded the control group’s. The differentiation between groups was particularly pronounced in self-repair behavior—taking the English-to-Chinese direction as an example, the experimental group’s repair frequency decreased by 28% (from 3.79 times/min to 2.72 times/min), while the control group’s repair frequency increased by 35% (from 3.06 times/min to 4.14 times/min), with the two groups showing opposite change directions.

Viewed from the perspective of the dual mechanisms cognitive control framework, the experimental group’s systematic decline in paralinguistic indicators can be interpreted as a strategic shift from reactive to proactive control—proactive control reduces the need for post-hoc repairs by activating goal representations in advance, thereby decreasing the occurrence frequency of reactive markers such as pauses, silences, and self-repairs. The control group’s negative changes on some indicators (such as the 35% increase in self-repair frequency) suggest that in the absence of targeted training, individuals facing cognitive challenges tend to intensify reactive control strategies. The above findings provide preliminary support for H1 (paralinguistic features as behavioral markers of cognitive control strategies) and H2 (metacognitive training promoting cognitive control strategy shifts).

### 3.2 Within-group changes in paralinguistic features

Within-group pre-post comparison results revealed systematic differences in paralinguistic feature changes between the two groups (Table 4). Regarding lengthening features, the experimental group reached highly significant levels in both task directions, with effect sizes reaching the large effect level in the Chinese-to-English direction (*r* = 0.77); the control group showed significant change only in the Chinese-to-English direction, with effect size (*r* = 0.42) approximately half that of the experimental group. The difference was particularly pronounced in the English-to-Chinese direction: the experimental group’s lengthening frequency dropped from 1.54 times/min to 0.00 times/min, while the control group showed no significant change. As a typical marker of lexical retrieval difficulty during speech production (Kormos, 2006), the elimination of lengthening suggests the experimental group may have achieved a strategic shift from reliance on real-time remediation (reactive control) to prospective planning (proactive control).

**Table 4:** Results of within-group comparisons for key paralinguistic variables.

Variable	Direction	Experimental group			Control group		
		z/t	*p*	r/d	z/t	*p*	r/d
Lengthening (E-C)	T1 > T2	−2.56	0.010*	0.47	−1.63	0.104	0.30
Lengthening (C-E)	T1 > T2	−4.23	<0.01**	0.77	−2.30	0.021*	0.42
Pause frequency (E-C)	T1 > T2	−2.75	0.005**	0.50	−0.89	0.374	0.16
Pause frequency (C-E)	T1 > T2	−2.31	0.021*	0.42	−1.02	0.308	0.19
Pause duration (E-C)	T1 = T2	−0.46	0.643	0.08	−1.76	0.079	0.32
Pause duration (C-E)	T1 = T2	−0.69	0.491	0.13	−2.54	0.011*	0.46
Silence frequency (E-C)	T1 > T2	−4.21	<0.01**	0.77	3.52	0.001**	0.64
Silence frequency (C-E)	T1 > T2	4.45	<0.01**	0.81	2.00	0.055	0.74
Self-repair (E-C)	T1 > T2	3.07	0.005**	0.56	−1.45	0.158	0.27
Self-repair (C-E)	T1 = T2	−0.13	0.894	0.02	−3.23	0.027*	0.59

Direction of change: T1 > T2 indicates decrease; T1 = T2 indicates no significant change. *indicates *p* < 0.05; **indicates *p* < 0.01.

**Figure 3:**
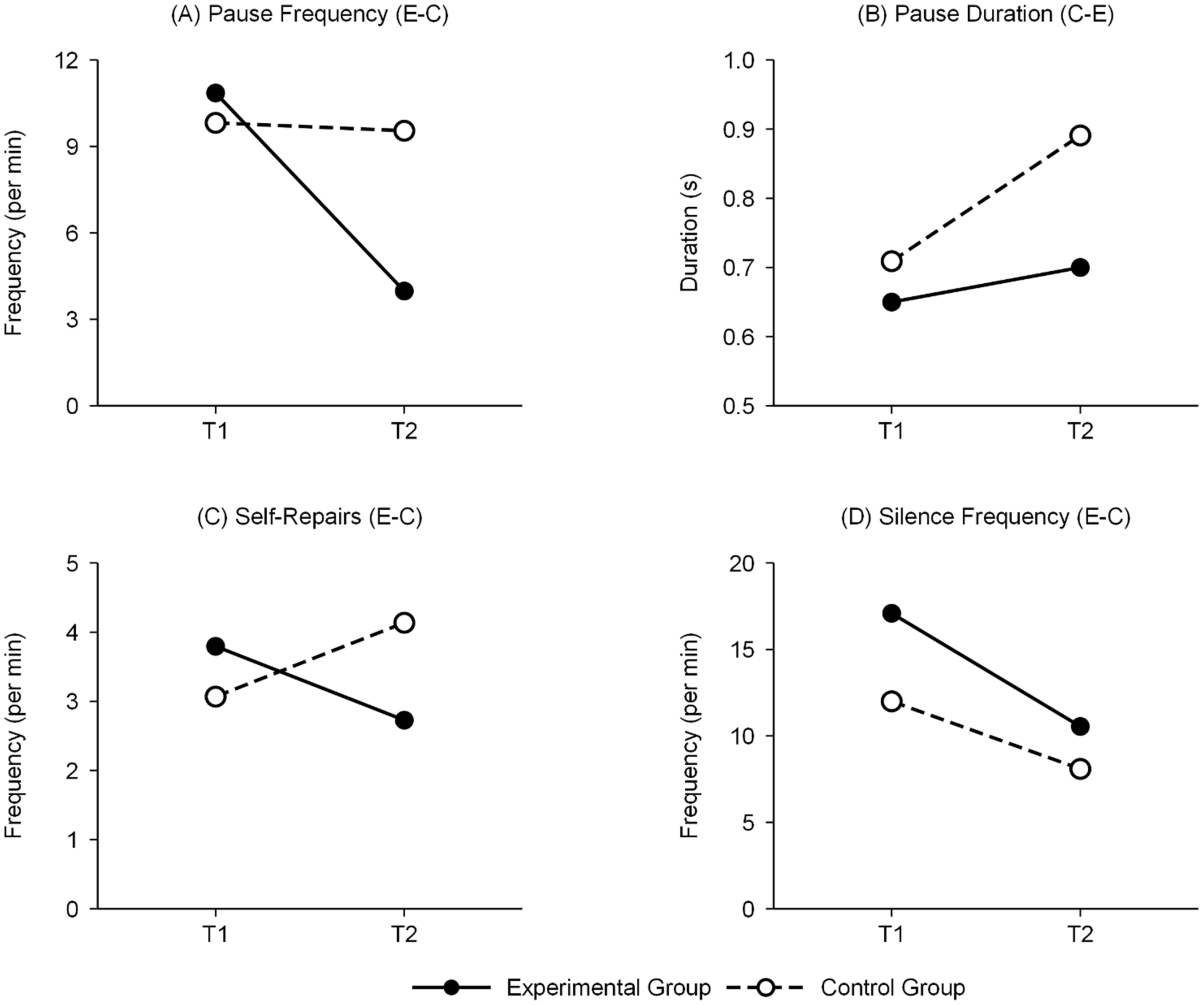
Interaction effects between group and time on selected paralinguistic variables.

Pause features presented a dissociated pattern of frequency and duration. On the frequency dimension, the experimental group significantly declined in both directions (English-to-Chinese: z = −2.75, *p* < 0.01, *r* = 0.50; Chinese-to-English: *z* = −2.31, *p* < 0.05, *r* = 0.42), with decreases of 63 and 43% respectively; the control group did not reach significance in either direction (Figure 3). On the duration dimension, the control group’s pause duration significantly extended (Chinese-to-English direction from 0.71 s to 0.89 s, a 25% increase), while the experimental group remained stable. This dissociated pattern carries important theoretical implications: the experimental group’s decrease in pause frequency while maintaining stable duration suggests their retained pauses serve more of a planning function (a characteristic of proactive control); the control group’s extended pause duration while frequency remained unchanged suggests their pauses serve more for post-hoc remediation (a characteristic of reactive control).

Regarding silence indicators, the experimental group showed significant improvement in both directions, with effect sizes reaching the large effect level (English-to-Chinese: *r* = 0.77; Chinese-to-English: *d* = 0.81). The control group also reached significance in the English-to-Chinese direction (*d* = 0.64), but only marginally significant in the Chinese-to-English direction (*p* = 0.055). The experimental group showed a larger decrease in the English-to-Chinese direction, bringing the two groups closer at post-test (post-test: 10.51 times/min vs. 8.05 times/min). It should be noted that the experimental group’s baseline silence frequency exceeded the control group’s (17.09 times/min vs. 11.96 times/min, 43% numerical difference). Although this baseline difference did not reach statistical significance, it may somewhat affect interpretation of improvement magnitude. Despite the baseline difference, the experimental group’s decrease (38.5%) still significantly exceeded the control group’s (32.7%), and interaction effect analysis did not show significant interaction effects for silence frequency in the English-to-Chinese direction, suggesting that baseline differences had relatively limited impact on intervention effect evaluation.

Self-repair behavior changes showed opposite trajectories between the two groups, a phenomenon providing key evidence for the dual mechanisms framework. The experimental group’s repair frequency significantly declined in the English-to-Chinese direction [*t*(29) = 3.07, *p* < 0.01, *d* = 0.56], with input-driven repairs decreasing notably (*z* = −3.11, *p* < 0.01, *r* = 0.57); interpreter-driven repairs in the Chinese-to-English direction dropped from 0.83 times/min to zero (*z* = −2.84, *p* < 0.01, *r* = 0.52). The control group showed the opposite pattern: repair frequency in the Chinese-to-English direction significantly increased [*t*(29) = −3.23, *p* < 0.05, *d* = 0.59], with interpreter-driven repairs rising from zero to 1.07 times/min (*z* = −3.00, *p* < 0.01, *r* = 0.55). Examining repair type distributions, the experimental group’s input-driven repair proportion decreased from approximately 75% to approximately 65%, while the control group’s interpreter-driven repair proportion rose from approximately 5% to approximately 20%. According to Tang (2020) classification framework, interpreter-driven repairs primarily serve to gain cognitive processing time; their increased proportion reflects the control group’s deepening reliance on reactive control strategies.

Paralinguistic differentiators also showed between-group differences: the experimental group showed declining trends in both directions (English-to-Chinese: 11 → 6; Chinese-to-English: 12 → 8), with cross-directional consistency in changes; the control group exhibited inconsistency across directions (English-to-Chinese: 7 → 7; Chinese-to-English: 11 → 8).

Within-group change results support H2’s prediction: metacognitive training can promote systematic improvement in paralinguistic indicators, an improvement interpretable as a shift in cognitive control strategies from reactive to proactive. The control group’s negative changes on some indicators further support H1, indicating that paralinguistic features can sensitively reflect individuals’ cognitive control strategy states.

### 3.3 Interpreting performance and interaction effects

Within-group comparisons of task performance showed that both groups exhibited significant improvement, but with systematic differences in improvement magnitude (Table 5). On accuracy indicators, improvement magnitudes were relatively similar between groups, with the experimental group averaging approximately 2 percentage points higher than the control group (22 percentage points vs. 20 percentage points). On completeness indicators, the between-group gap widened notably, with the experimental group’s average decrease at 37.5 percentage points compared to the control group’s 25.5 percentage points—the former approximately 1.5 times the latter—suggesting that metacognitive training’s facilitative effect on information transmission completeness exceeds its effect on accuracy.

**Table 5:** Interpreting performance by group and direction.

Measure	Direction	Group	T1 (Mdn)	T2 (Mdn)	Change	*z*	*p*
Words correctly interpreted (%)	E-C	Experimental	27	49	+22	−3.53	<0.01
		Control	34	54	+20	−3.24	<0.01
	C-E	Experimental	53	73	+20	−3.86	<0.01
		Control	59	72	+13	−2.42	<0.05
Meaning groups uninterpreted (%)	E-C	Experimental	82	41	−41	−4.21	<0.01
		Control	69	44	−25	−3.70	<0.01
	C-E	Experimental	54	20	−34	−4.72	<0.01
		Control	43	20	−28	−3.82	<0.01

*N* = 60 (*n* = 30 per group). Mdn = median. E-C = English to Chinese; C-E = Chinese to English. Change = T2 − T1. *p < 0.05. **p < 0.01.

Mixed-design repeated measures ANOVA interaction effect tests provided statistical validation for the above observations (Table 6). For task performance indicators, the group × time interaction effect for percentage of untranslated meaning units reached significance in both task directions [English-to-Chinese: *F*(1, 58) = 9.67, *p* = 0.003, η^2^p = 0.14; Chinese-to-English: *F*(1, 58) = 4.58, *p* = 0.036, η^2^p = 0.07]. The interaction effect for percentage of correctly translated vocabulary reached significance only in the Chinese-to-English direction [*F*(1, 58) = 5.12, *p* = 0.027, η^2^p = 0.08], not reaching significance in the English-to-Chinese direction [*F*(1, 58) = 0.89, *p* = 0.349, η^2^p = 0.02].

**Table 6:** Mixed-design ANOVA results: Group × Time interaction effects.

Variable	Direction	*F*(1, 58)	*p*	η^2^p
Words correctly interpreted (%)	E-C	0.89	0.349	0.02
	C-E	5.12	0.027*	0.08
Meaning groups uninterpreted (%)	E-C	9.67	0.003**	0.14
	C-E	4.58	0.036*	0.07
Pause frequency	E-C	8.23	0.006**	0.12
	C-E	5.87	0.019*	0.09
Silence frequency	E-C	2.94	0.092	0.05
	C-E	6.35	0.015*	0.10
Self-repair frequency	E-C	12.41	0.001**	0.18
	C-E	9.18	0.004**	0.14

For paralinguistic feature indicators, pause frequency interaction effects reached significance in both directions (English-to-Chinese: η^2^p = 0.12; Chinese-to-English: η^2^p = 0.09). Self-repair frequency interaction effects reached the large effect size level in both directions [English-to-Chinese: *F*(1, 58) = 12.41, *p* = 0.001, η^2^p = 0.18; Chinese-to-English: *F*(1, 58) = 9.18, *p* = 0.004, η^2^p = 0.14], statistically validating the opposite change trajectories in self-repair behavior between the two groups. This interaction effect pattern provides behavioral-level support for the dual mechanisms cognitive control framework: the experimental group’s decrease in self-repair frequency reflects enhanced proactive control (reducing the need for post-hoc repairs through advance planning), while the control group’s increase in self-repair frequency reflects intensified reactive control (addressing cognitive challenges through increased post-hoc repairs). Silence frequency interaction effects showed directional differences—reaching significance in the Chinese-to-English direction [*F*(1, 58) = 6.35, *p* = 0.015, η^2^p = 0.10], marginally significant in the English-to-Chinese direction [*F*(1, 58) = 2.94, *p* = 0.092, η^2^p = 0.05].

Joint analysis of baseline levels and change magnitudes revealed the existence of catch-up effects. Taking the Chinese-to-English direction as an example, the experimental group’s percentage of correctly translated vocabulary shifted from trailing the control group by 6 percentage points (53% vs. 59%) to leading by 1 percentage point at T2 (73% vs. 72%). The moderating effect of task direction on training effects is evident from cross-directional comparisons of interaction effects: between-group differences in the Chinese-to-English direction (accuracy interaction effect η^2^p = 0.08) exceeded those in the English-to-Chinese direction (η^2^p = 0.02). From a cognitive load theory perspective, the Chinese-to-English direction places higher demands on cognitive resources, and differences in cognitive control strategies become more fully manifest in that direction—a finding consistent with Braver (2012) prediction that proactive control advantages become more pronounced under high cognitive load conditions.

The above interaction effect test results support H3’s prediction: metacognitive training improvement effects significantly exceed conventional training. The large effect size interaction effects for self-repair frequency (η^2^p = 0.14–0.18) provide behavioral-level empirical support for the applicability of Braver’s dual mechanisms framework in complex speech production, validating the trainability of cognitive control strategy shifts from reactive to proactive.

## 4 Discussion

This study systematically examined the effects of metacognitive training on paralinguistic features in complex speech production tasks through a randomized controlled trial. Mixed-design ANOVA interaction effect test results provided statistical-level support for the three research hypotheses. The findings offer behavioral-level evidence for the applicability of Braver (2012) Dual Mechanisms of Cognitive Control framework in speech production and reveal the trainability of cognitive control strategy shifts from reactive to proactive.

The experimental group showed significant improvement across core paralinguistic indicators including lengthening, pause frequency, silence frequency, and self-repair frequency, while the control group exhibited negative changes on some indicators—the statistical significance of this differentiated pattern has been confirmed through interaction effect tests. The group × time interaction effect for self-repair frequency reached highly significant levels in both task directions, with effect sizes at the large effect level (η^2^p = 0.14–0.18). This finding resonates with Plevoets and Defrancq (2018) large-scale corpus study, which found that cognitive load indicators significantly predicted the occurrence frequency of disfluency markers in speech production. Betz et al. (2023) experimental research further confirmed the causal relationship between cognitive load and speech hesitation. Building on this foundation, the present study demonstrates that paralinguistic disfluency phenomena are not merely passive reflections of cognitive load but can be actively regulated through targeted metacognitive training—a finding that extends theoretical understanding of the nature of paralinguistic features, from mere cognitive state indicators to malleable cognitive control skills.

The control group’s extended pause duration (25% increase) and increased self-repair frequency (35% increase) reveal a noteworthy phenomenon. In the absence of metacognitive awareness intervention, individuals facing high cognitive load tasks tend to passively rely on paralinguistic means to gain processing time rather than actively optimizing speech planning processes. Viewed from Braver (2012) dual mechanisms framework perspective, this change pattern reflects the control group’s deepening reliance on reactive control strategies—reactive control is characterized by post-hoc detection and repair, mobilizing cognitive resources to respond only after problems emerge. Mäki-Marttunen et al. (2019) fMRI study provided direct evidence for the neural mechanisms of reactive control, finding that activation patterns in prefrontal cortex and brainstem regions under high task context load conditions match characteristics of reactive control. Bugg (2014) research from a developmental perspective showed that reactive control is relatively preserved during aging, suggesting that the two control modes may rely on different neurocognitive mechanisms. The change pattern exhibited by the control group in this study aligns with the above findings, while the experimental group successfully achieved a strategic shift from passive response to active regulation through metacognitive training.

Between-group differences in lengthening features provide further evidence for the applicability of the dual mechanisms framework. The experimental group’s lengthening frequency dropped from baseline (1.54 times/min) to zero in the English-to-Chinese direction, contrasting with the control group’s non-significant change. It should be noted that the zero value here is the median, indicating that at least half of experimental group members showed no lengthening at post-test, but not that all individuals completely eliminated it. This group-level significant improvement may be influenced by multiple factors: the experimental materials were standardized speech texts with controlled lexical density and syntactic complexity, providing more adequate conditions for prospective planning compared to spontaneous conversation; English-to-Chinese, as the native language output direction, involves lower cognitive load for lexical retrieval; the 12-week systematic training prompted some individuals to achieve strategy shifts under these task conditions. As a typical marker of lexical retrieval difficulty (Kormos, 2006), the substantial group-level reduction in lengthening suggests the experimental group may have achieved a cognitive control strategy shift from real-time remediation to prospective planning, consistent with Braver et al. (2021) findings that proactive control optimizes processing efficiency by activating goal representations in advance. Research by Gonthier et al. (2016) and Snijder et al. (2023) further supports the trainability and separability of cognitive control strategies; the present study extends this conclusion to complex speech production contexts.

The dissociated pattern of frequency and duration in pause features carries important theoretical implications. The experimental group’s significant decrease in pause frequency (43–63%) while duration remained stable, versus the control group’s unchanged frequency while duration significantly extended (25% increase). Han and An (2021) research on pause measurement thresholds suggested functional heterogeneity in pause phenomena, and the present findings resonate with this. From the dual mechanisms framework perspective, the experimental group’s retained pauses serve more of a planning function, reflecting characteristics of proactive control; the control group’s extended pause duration suggests their pauses serve more for *post-hoc* remediation, reflecting characteristics of reactive control. Bartłomiejczyk and Gumul (2024) pointed out that different types of disfluency phenomena reflect different levels of cognitive processing; the present findings provide supporting evidence from a training intervention perspective, further revealing that metacognitive training can selectively reduce hesitation pauses while preserving planning pauses.

Between-group changes in self-repair behavior provide more refined clues for understanding the mechanisms of cognitive control strategy shifts. According to Tang (2020) classification framework, input-driven repairs stem from perception of gaps between output and targets, while interpreter-driven repairs serve to gain cognitive processing time. The experimental group’s synchronized reduction in both repair types reflects a dual improvement effect: metacognitive training both enhanced information conversion efficiency (reducing input-driven repairs) and strengthened prospectiveness in speech planning (reducing interpreter-driven repairs). The control group’s interpreter-driven repair proportion rose from approximately 5% to approximately 20%, suggesting their increased reliance on repair strategies to alleviate processing pressure when facing cognitive challenges. This finding theoretically resonates with Oliveira et al. (2023) research on the relationship between processing fluency and cognitive control: when individuals lack active regulatory capacity over cognitive processes, they more easily fall into processing modes dominated by reactive control.

The moderating effect of task direction on training effects is evident from cross-directional differences in interaction effects. Between-group differences in the Chinese-to-English direction (accuracy interaction effect η^2^p = 0.08) exceeded those in the English-to-Chinese direction (η^2^p = 0.02), while the English-to-Chinese direction showed larger interaction effects on completeness indicators (η^2^p = 0.14) than the Chinese-to-English direction (η^2^p = 0.07). Lin et al. (2018) research found that working memory can explain 50–51% of speech disfluency variance; the present study reveals another improvement pathway from a training intervention angle—optimizing the allocation efficiency of cognitive control strategies by enhancing metacognitive awareness.

Analyzing from a theoretical mechanism perspective, the experimental group’s systematic improvement in paralinguistic features may stem from enhanced metacognitive awareness. Flavell (1979) theoretical framework on metacognitive monitoring promoting cognitive regulation suggests that training may enhance individuals’ metacognitive awareness of paralinguistic features, thereby optimizing cognitive control strategy selection. Roebers (2017) framework integrating executive function and metacognition indicates that metacognitive monitoring and executive control share some cognitive resources, helping explain how metacognitive training in this study was able to influence cognitive control strategy selection. Boldt and Gilbert (2022) neuroimaging research revealed partially overlapping yet separable neural bases for metacognitive monitoring and metacognitive control; future research could employ standardized metacognitive assessment tools combined with neuroimaging techniques to directly test the mediating role of metacognitive awareness in cognitive control strategy shifts.

While no prior studies have directly tested the link between specific paralinguistic training tasks and individual WM components, a speculative mapping can be proposed on theoretical grounds. The reduction in lengthening frequency may reflect enhanced phonological loop efficiency, as lengthening is a recognized marker of lexical retrieval difficulty during phonological encoding (Kormos, 2006). The decrease in pause frequency aligns with improved central executive updating capacity, consistent with Oberauer (2019) characterization of the central executive as responsible for maintaining and updating goal representations during ongoing processing. The decline in self-repair frequency suggests strengthened episodic buffer integration, the component Baddeley (2000) identified as binding cross-modal information into coherent representations. This pattern is consistent with the WM components identified by Mellinger and Hanson (2021) and Dong et al. (2018) as most predictive of and responsive to interpreting training. These proposed correspondences remain speculative and await direct empirical validation through studies that concurrently measure specific WM components and paralinguistic training outcomes.

The above findings carry implications for extending theoretical frameworks in cognitive psychology. By introducing complex speech production as a high ecological validity task context, this study extends the applicability boundaries of Braver’s dual mechanisms cognitive control framework from discrete-trial laboratory tasks to continuous, dynamic cognitive processing. Dong and Li (2021) research on attentional control emphasized the importance of dynamic cognitive resource allocation; the present study further reveals the role of metacognitive training in optimizing this allocation process. Hervais-Adelman and Babcock (2021) explored the coordinated operation of multiple cognitive systems in extreme cognitive control contexts from a neurobiological perspective; the behavioral data from this study provide complementary evidence from a training intervention perspective for this neural mechanism model.

Several limitations of this study warrant mention. Regarding sample characteristics, research participants presented high homogeneity: all were third-year undergraduates from a single university’s School of Foreign Languages, aged 20–22, all native Chinese speakers with English as L2 and no prior interpreting training experience. While this homogeneity enhances internal validity, it limits external validity of conclusions. The applicability of training effects remains unclear for the following groups: interpreting learners from different educational backgrounds (such as graduate students or working interpreters), different age groups (such as middle-aged or older learners), other language pair combinations (such as non-English-Chinese bilinguals), individuals with prior interpreting experience, and non-student populations whose cognitive control strategies may differ. Sample size (*n* = 30 per group), though meeting statistical power requirements, limits in-depth exploration of individual differences. Regarding research design, although random assignment was employed and blinding was achieved at data rating and annotation stages (raters and annotators were blind to group assignment), due to the nature of the intervention, experimenters and participants could not remain blind to group assignment. Participants knowing which training they received may have led the experimental group to produce Hawthorne effects and the control group to exhibit compensatory effort or negative reactions, thereby affecting the authenticity of intervention effects. At the measurement level, the lack of standardized metacognitive assessment tools and neuroimaging data limits exploration of underlying mechanisms. Regarding study duration, long-term maintenance of training effects and cross-task transfer await examination. Regarding statistical analysis, this study did not correct for multiple statistical tests, which may increase Type I error risk. Although main conclusions were based on theoretically predicted interaction effect tests, and all significant results were accompanied by medium to large effect sizes, multiple testing issues still warrant caution. Future research could consider adopting more stringent significance thresholds or False Discovery Rate (FDR) correction methods, maintaining adequate statistical power while controlling Type I error. Additionally, speech rate (words per minute) was not measured or incorporated as a statistical covariate. Because paralinguistic frequency indicators are expressed as counts per minute, changes in overall speaking rate could partially account for observed frequency reductions independently of cognitive control strategy shifts; future studies should measure speech rate and include it as a covariate to disambiguate production-rate effects from genuine cognitive control changes. Furthermore, the inference from paralinguistic behavioral markers to cognitive control modes (proactive versus reactive) remains indirect and theory-driven; no independent cognitive control measures—such as the AX-CPT task with contextual cueing, which is canonical in DMC research—were administered pre- or post-training to provide convergent validation of the proposed control-shift mechanism. Future studies should incorporate standard DMC paradigms alongside paralinguistic annotation to directly test the theoretical link. Finally, the experimental group received additional training content, individualized instructor feedback, and novel task materials beyond the control group’s standard instruction, introducing potential expectancy and Hawthorne effects that cannot be fully disentangled from metacognitive training effects per se. Future studies should employ an active control condition matched for total contact time, instructor interaction, and task novelty to isolate the specific contribution of paralinguistic cognitive training.

Future research could extend the present findings from multiple directions. At the theoretical validation level, an important research direction is examining whether the cognitive control strategy shifts found in this study can be replicated in other complex cognitive tasks. Kassai et al. (2019) meta-analysis of executive function training transfer effects showed relatively robust near-transfer effects but relatively limited far-transfer effects, suggesting future research needs to systematically examine the transfer boundaries of metacognitive training effects. Simons et al. (2016) comprehensive review of brain training research pointed out that generalizability of cognitive training effects is a core issue in the field. Gobet and Sala (2023) pointed out methodological problems existing in the cognitive training field from a critical perspective, problems that warrant full consideration in future research designs. At the task context level, research could extend to other complex tasks requiring real-time cognitive control, such as impromptu speaking, debate, multi-task coordination, and complex decision-making, examining the generalizability of paralinguistic features as markers of cognitive control strategies. At the neural mechanism level, event-related potentials or functional near-infrared spectroscopy and other neuroimaging techniques could be employed to directly examine the effects of metacognitive training on prefrontal cortex activity patterns. Etzel et al. (2022) dual mechanisms cognitive control dataset provides methodological reference for this research direction. At the individual differences level, further examination of how cognitive traits such as working memory capacity and executive function efficiency moderate training effects could explore boundary conditions for cognitive control strategy plasticity. At the applied extension level, examining the potential benefits of metacognitive training for other populations with cognitive control demands (such as individuals with attention deficit hyperactivity disorder or older adults with cognitive decline) could explore the clinical translation value of cognitive control interventions.

## 5 Conclusion

This study employed a randomized controlled trial design to systematically examine the effects of paralinguistic cognitive training on consecutive interpreting performance and paralinguistic output among 60 student interpreters through 13-week longitudinal tracking, providing empirical support for the applicability of Braver’s Dual Mechanisms of Cognitive Control framework in complex speech production contexts. Results indicate that paralinguistic features can sensitively reflect differences in cognitive control strategies. The control group’s negative changes—pause duration extending from 0.71 to 0.89 s and self-repair frequency increasing by 35%—embody speech production characteristics under reactive control dominance, while the experimental group’s systematic improvements across core indicators including pause frequency (63% decrease), lengthening frequency (dropping to zero in the English-to-Chinese direction), and self-repair frequency (28% decrease) reflect successful transformation toward proactive control strategies. Mixed-design ANOVA interaction effect tests confirmed that the experimental group’s improvement magnitude significantly exceeded the control group’s, with interaction effects for self-repair frequency particularly prominent (English-to-Chinese: η^2^p = 0.18; Chinese-to-English: η^2^p = 0.14), and interaction effects for percentage of untranslated meaning units reaching significance in both directions (English-to-Chinese: η^2^p = 0.14; Chinese-to-English: η^2^p = 0.07). The scissor-pattern differentiation between the two groups on self-repair indicators provides behavioral-level evidence for the cognitive control strategy plasticity hypothesis. This study’s theoretical contribution to cognitive psychology lies in revealing the validity of paralinguistic features as overt indicators of cognitive control strategies and confirming the feasibility of promoting individuals’ shift from reactive to proactive control through metacognitive training, thereby extending the application boundaries of the dual mechanisms cognitive control framework. At the practical level, results indicate that paralinguistic cognition, as a cultivable metacognitive ability, can be effectively enhanced through systematic training, providing a new entry point for intervention research on cognitive control ability. Although this study employed random assignment to control selection bias, limitations remain including relatively limited sample size and absence of double-blind design. Future research could employ event-related potentials or functional near-infrared spectroscopy and other neuroimaging techniques to deeply explore the neural mechanisms by which paralinguistic cognitive training promotes cognitive control strategy shifts.

## Data Availability

The raw data supporting the conclusions of this article will be made available by the authors, without undue reservation.
