# Supplementary material for: Pauses, silences, and cognitive control: psycholinguistic markers of speech planning in interpreter training
Source: Front Psychol. 2026 May 19;17:1775771. doi: 10.3389/fpsyg.2026.1775771 (PMC13226530; doi:10.3389/fpsyg.2026.1775771)
Supplement: Supplementary file 1 [file Table_1.DOCX]

**Supplementary File S1**

**Sample Training Materials for the Paralinguistic Cognitive Training Program**

*Pauses, Silences, and Cognitive Control: Psycholinguistic Markers of Speech Planning
in Interpreter Training*

**Overview of the Training Program**

The paralinguistic cognitive training program was designed based on Flavell's (1979) metacognition theory, Braver's (2012) Dual Mechanisms of Cognitive Control (DMC) framework, and Kormos's (2006) speech production model. It spanned 12 weeks and comprised four sequential modules, each targeting specific cognitive mechanisms identified as critical to the proactive–reactive control transition. Training followed a blended learning format: weekly materials (theoretical explanations, annotated transcripts, practice audio) were released via an online platform, and participants submitted weekly practice outputs for individualized feedback from the research team.

**Table S1. Overview of the four training modules.**

| **Module** | **Focus** | **Duration** | **Proposed Cognitive Mechanism** |
| --- | --- | --- | --- |
| 1 | Speech Rate and Prosody Control | Weeks 1–3 | Phonological loop rehearsal; attentional monitoring of articulatory output |
| 2 | Pauses and Silences | Weeks 4–6 | Central executive proactive allocation; self-monitoring capacity |
| 3 | Self-Repair | Weeks 7–9 | Episodic buffer integration; metacognitive error detection and self-correction |
| 4 | Paralinguistic Differentiators | Weeks 10–12 | Attentional filtering; controlled processing of non-verbal signals |

All sample tasks below use materials from the study's AI-themed stimulus corpus: Elon Musk's speech at the 2023 World Artificial Intelligence Conference (English source) and Huawei rotating chairman Hu Houkun's speech at the same event (Chinese source). Excerpts were adapted for training purposes to match the difficulty level of each module.

**Module 1: Speech Rate and Prosody Control (Weeks 1–3)**

**1.1 Theoretical Background**

Fluent speech production requires dynamic management of speech rate and prosodic features. Within the DMC framework, proactive control is characterized by planned, pre-emptive rate adjustments at upcoming information boundaries, whereas reactive control manifests as abrupt rate changes triggered by encountered difficulty. This module trains learners to consciously regulate rate and pitch to convey semantic functions (e.g., emphasis, contrast, topic shift) while simultaneously alleviating phonological loop load through prospective planning.

**1.2 Annotation Symbols**

**Table S1-1. Prosodic annotation symbols used in Module 1 exercises.**

| **Symbol** | **Meaning** |
| --- | --- |
| → | Speed up (background/connective information) |
| ← | Slow down (key concept / emphasis) |
| / | Brief planning pause (≥ 0.2 s, at syntactic boundary) |
| // | Long planning pause (≥ 2.0 s, clause boundary) |
| _ | Syllable stress / pitch rise |
| [ ] | Pitch boundary (intonation unit) |

**1.3 Sample Task 1-A: Pre-Interpretation Prosody Marking**

***Instructions***

Read the source excerpt below carefully. Before interpreting, mark the text using the symbols above to plan your prosodic delivery. Then record your consecutive interpretation and compare your actual output with your pre-interpretation marks.

***Source Text (English → Chinese, ~60 words)***

*"The potential of artificial intelligence to transform our world is extraordinary. We stand at an inflection point—the decisions we make today will determine the trajectory of this technology for generations to come. At Tesla and SpaceX, AI is already accelerating innovation at a pace that was previously unimaginable. But I want to be very clear: development of AI must be guided by safety and responsibility."*

***Annotated Model Example***

*[The potential of AI to transform our world] ← is _extraordinary. // [We stand at an inflection point]—/ [the decisions we make today] ← will determine / [the trajectory of this technology] → for generations to come. // [At Tesla and SpaceX,] → AI is already accelerating innovation / at a pace → that was previously unimaginable. // But I want to be _very clear: // [development of AI] ← must be guided / by _safety and _responsibility.*

*Note: ← marks signal proactive slowing for key concepts; // marks pre-planned long pauses at sentence boundaries to allow note-checking; → marks allow faster delivery for background clauses, reducing phonological loop demand.*

**1.4 Sample Task 1-B: Rate Self-Monitoring Exercise**

***Instructions***

Listen to the model audio clip (Track M1-B). The speaker produces the same passage at three rates: (a) 80–100 wpm, (b) 130–150 wpm, (c) 180+ wpm. Answer the following reflection questions and submit your written responses:

(1) At which rate does the speaker's prosodic clarity deteriorate most noticeably? Identify the specific location (sentence / clause) where breakdown occurs.
(2) At which rate are semantic boundaries most clearly signaled? Provide two examples.
(3) Record yourself interpreting the passage at a self-chosen optimal rate. Annotate your own output and identify two locations where your rate was reactive rather than planned.

**Module 2: Pauses and Silences (Weeks 4–6)**

**2.1 Theoretical Background**

Module 2 targets the distinction between hesitation pauses—reactive responses to processing difficulty occurring mid-phrase or co-occurring with filled pauses—and planning pauses—proactive insertions at syntactic boundaries that facilitate prospective information organization. Learners train central executive self-monitoring by learning to (a) recognize the functional difference between the two pause types in model outputs, (b) eliminate hesitation pauses from their own production, and (c) deploy planning pauses strategically as information organization tools.

**2.2 Pause Classification Criteria**

**Table S2-1. Operational criteria for pause classification in Module 2.**

| **Pause Type** | **Position** | **Co-occurring Features** | **Cognitive Function** |
| --- | --- | --- | --- |
| Planning pause (P) | Major syntactic boundary (clause / sentence juncture) | No filled pause; precedes new propositional unit | Proactive: prospective preparation for upcoming segment |
| Hesitation pause (H) | Mid-phrase / mid-clause | Often co-occurs with uh/um, false starts, or restarts | Reactive: triggered by encountered processing difficulty |

**2.3 Sample Task 2-A: Pause Classification**

***Instructions***

Read the transcript below of a student's E-C consecutive interpretation output. Each marked pause includes its duration. Classify each pause as P (planning) or H (hesitation) using the criteria in Table S2-1, and provide a brief justification.

***Student Output Transcript (with pauses marked)***

*人工智能改变世界的潜力 [0.4s-1] 是非凡的。 [1.8s-2] 我们正站在一个拐点——[0.3s-3] 也就是说 [0.6s-4] 今天我们 [0.5s-5] 我们做出的决策 [1.5s-6] 将决定这项技术未来几代人的发展轨迹。 [2.1s-7] 在特斯拉和太空探索技术公司，[0.8s-8] 人工智能已经在以 [0.4s-9] 前所未有的速度 [1.0s-10] 加速推动着创新。 [2.3s-11]*

***Answer Key***

**Table S2-2. Answer key for Task 2-A.**

| **Pause #** | **Duration** | **Classification** | **Justification** |
| --- | --- | --- | --- |
| 1 | 0.4s | H | Mid-NP, before predicate; co-occurs with subsequent restart ("是非凡的") |
| 2 | 1.8s | P | End of first complete sentence; boundary before new topic clause |
| 3 | 0.3s | H | Mid-clause after dash; precedes reformulation marker "也就是说" |
| 4 | 0.6s | H | After reformulation; co-occurs with mid-phrase disfluency |
| 5 | 0.5s | H | Mid-NP; triggers self-repair (repeated subject "我们") |
| 6 | 1.5s | P | End of subject NP; boundary before verb phrase |
| 7 | 2.1s | P | End of first complete proposition; long inter-sentence pause |
| 8 | 0.8s | P | Clause boundary after PP adjunct; precedes main clause |
| 9 | 0.4s | H | Mid-AdvP; no syntactic boundary justification |
| 10 | 1.0s | P/H (ambiguous) | Occurs at AdvP boundary but follows slight vowel lengthening on "速度", suggesting possible retrieval difficulty; classification depends on whether prosodic context is prioritized over syntactic position |
| 11 | 2.3s | P | End of complete sentence; long inter-sentence boundary pause |

**2.4 Sample Task 2-B: Strategic Pause Insertion**

***Instructions***

Before interpreting the source text below (C-E direction), mark // at every major syntactic boundary where you plan to insert a deliberate planning pause. Then record your interpretation and evaluate: (1) Did you successfully insert pauses at your pre-marked locations? (2) Did any hesitation pauses appear at non-boundary positions? (3) How did pre-planning pause positions affect your output fluency?

***Source Text (Chinese → English, ~55 words)***

*"人工智能正在以前所未有的速度重塑全球产业格局。华为在过去几年里，持续加大对AI基础设施的投入。我们相信，AI的核心竞争力在于算力、算法和数据的协同创新。中国拥有全球最大的数字化应用场景，这为AI技术的落地提供了得天独厚的条件。"*

***Model Pre-Marked Version***

*人工智能正在以前所未有的速度重塑全球产业格局。// 华为在过去几年里，// 持续加大对AI基础设施的投入。// 我们相信，// AI的核心竞争力 / 在于算力、算法和数据的协同创新。// 中国拥有全球最大的数字化应用场景，// 这为AI技术的落地 / 提供了得天独厚的条件。//*

**Module 3: Self-Repair (Weeks 7–9)**

**3.1 Theoretical Background**

This module is based on Tang's (2020) three-category repair classification framework and targets episodic buffer integration and metacognitive self-correction. Learners train to (a) recognize the source and type of repair in model outputs, (b) identify the cognitive mechanism underlying each repair type, and (c) develop pre-emptive comprehension and production strategies to reduce the need for post-hoc reactive corrections.

**3.2 Repair Type Classification**

**Table S3-1. Tang's (2020) three-category repair classification (adapted).**

| **Repair Type** | **Trigger Source** | **Definition** | **Cognitive Mechanism** |
| --- | --- | --- | --- |
| Input-driven (I) | Source text comprehension | Interpreter corrects a mishearing, misunderstanding, or ambiguity in the source language input | Reactive retrieval from episodic buffer; phonological re-processing of source |
| Output-driven (O) | Target language production | Interpreter corrects an error in TL lexical choice, morphosyntax, or prosody | Reactive monitoring of own output; error detection via internal speech monitor |
| Interpreter-driven (T) | Interpreter's own decision | Interpreter voluntarily adds clarification, adjusts register, or inserts a cultural adaptation not present in source | Metacognitive strategic adjustment; proactive if planned, reactive if triggered by real-time ambiguity |

**3.3 Sample Task 3-A: Repair Identification and Classification**

***Instructions***

Read the CI output transcript below. Identify all repair instances (underlined), classify each as I (input-driven), O (output-driven), or T (interpreter-driven), and explain the likely cognitive trigger for each repair.

***Student Output Transcript (E-C direction)***

*人工智能... [0.6s] 人工智能改变 [REPAIR-1: 改变→推动] 世界的潜力是令人难以置信的... [1.1s] 令人惊叹的。 [REPAIR-2: 令人难以置信的→令人惊叹的] 我们正站在 [0.5s] 一个... [0.4s] 一个历史节点 [REPAIR-3: 节点→拐点] 上。 [2.0s] 在特斯拉和... [REPAIR-4: 停顿+重启] 在特斯拉和太空探索技术公司，人工智能已经在推动 [0.3s] 推动着创新——[0.8s] 而且 [REPAIR-5: 插入连接词] 速度令人叹为观止。*

***Answer Key***

**Table S3-2. Answer key for Task 3-A.**

| **Repair #** | **Type** | **Trigger / Explanation** |
| --- | --- | --- |
| REPAIR-1 | O (Output-driven) | Lexical replacement: '改变' (change) → '推动' (drive/propel); interpreter self-monitors TL lexical precision and substitutes a more semantically accurate verb |
| REPAIR-2 | O (Output-driven) | Register/style revision: '令人难以置信的' is colloquially negative-toned; '令人惊叹的' is more formally positive. Interpreter detects register mismatch in own output |
| REPAIR-3 | I (Input-driven) / O (Output-driven) | Terminological correction: 'inflection point' misrendered as '历史节点'; interpreter retrieves correct term '拐点' from memory after noting output error |
| REPAIR-4 | I (Input-driven) | Proper noun processing difficulty: 'SpaceX' requires Chinese rendering; interpreter pauses and restarts to retrieve '太空探索技术公司' |
| REPAIR-5 | T (Interpreter-driven) | Voluntary cohesion addition: '而且' inserted to improve TL discourse coherence between two clauses not explicitly connected in source |

**3.4 Sample Task 3-B: Repair Reduction Practice**

***Instructions***

Listen to source text Track M3-B (C-E, 70 words, AI theme). Before interpreting, complete the following pre-processing steps to minimize repairs:
(1) Note-taking preparation: identify technical terms likely to require rendering decisions.
(2) Anticipation: predict discourse structure and connectives from the opening clause.
(3) Record your interpretation. Then review the transcript and count: total repairs (a), input-driven (b), output-driven (c), interpreter-driven (d).
(4) Compare your counts with your baseline repair frequency from Module 2 practice.
Target: reduce total repair frequency relative to your Week 4 baseline recording.

**Module 4: Paralinguistic Differentiators (Weeks 10–12)**

**4.1 Theoretical Background**

Paralinguistic differentiators are non-speech sounds produced during speech output, including laughter, coughing, throat-clearing, and sighing (Trager, 1958). In interpreting contexts, these sounds may serve communicative functions (e.g., turn-holding, hedging, rapport signaling) or reflect involuntary physiological responses (e.g., throat-clearing from dryness, nervous laughter under cognitive load). This module trains attentional filtering—the controlled processing capacity to distinguish and appropriately manage these signals. Proactive control involves planning the use of functional differentiators; reactive control manifests as uncontrolled production of non-functional differentiators under load.

**4.2 Classification of Paralinguistic Differentiators**

**Table S4-1. Classification of paralinguistic differentiators in interpreting contexts.**

| **Category** | **Type** | **Examples** | **Function** |
| --- | --- | --- | --- |
| Functional (F) | Turn-holding | Prolonged 'mm', 'uh' at clause boundaries | Signal to audience that interpretation is ongoing; prevent interruption |
| Functional (F) | Hedging/qualification | Quiet laugh before uncertain rendering | Signal approximate or uncertain interpretation |
| Functional (F) | Register alignment | Mirroring speaker's laughter at humorous remark | Preserve pragmatic tone of source speech |
| Non-functional (NF) | Physiological | Involuntary throat-clearing, coughing, dry-mouth sounds | No communicative function; may disrupt listener comprehension |
| Non-functional (NF) | Anxiety-driven | Nervous laughter mid-clause, audible breathing under load | Reflects cognitive overload; reactive response to difficulty |

**4.3 Sample Task 4-A: Functional vs. Non-Functional Classification**

***Instructions***

Read the annotated CI output transcript below. Each paralinguistic sound is marked in brackets. Classify each as F (functional) or NF (non-functional) and provide a brief rationale.

***Student Output Transcript (E-C, with paralinguistic sounds annotated)***

*[throat-clear-1] 人工智能... 推动世界变革的潜力是 [throat-clear-2] 非凡的。 [laugh-1] 我们正处于一个关键的历史节点。 [audible-inhale-1] 在特斯拉和太空探索技术公司，人工智能已经在 [mm-1] ——在以前所未有的速度推动着创新。 [laugh-2] 但我想非常明确地指出 [throat-clear-3]：人工智能的发展必须以安全和责任为导向。*

***Answer Key***

**Table S4-2. Answer key for Task 4-A.**

| **Sound** | **Classification** | **Rationale** |
| --- | --- | --- |
| throat-clear-1 | NF | Occurs at utterance onset before any content; likely nervous tension, no communicative value |
| throat-clear-2 | NF | Interrupts NP mid-production; physiological, disrupts fluency |
| laugh-1 | F (borderline) | May mirror source speaker's tone; however, if not present in source, classify NF (anxiety response to note review) |
| audible-inhale-1 | NF | Audible breath at clause boundary; physiological, reactive to cognitive load |
| mm-1 | F | Turn-holding 'mm' at false start; signals to listener that interpretation continues; prevents interruption—functional hesitation marker |
| laugh-2 | NF | Nervous laugh before challenging direct-address clause; reactive, non-communicative |
| throat-clear-3 | NF | Post-clause throat-clear; physiological, no discourse function |

**4.4 Sample Task 4-B: Self-Monitoring and Reduction Practice**

***Instructions***

Record your interpretation of source text Track M4-B (C-E, 65 words, AI theme). Transcribe your output and annotate all paralinguistic sounds. Then:
(1) Count: total paralinguistic sounds, functional (F), non-functional (NF).
(2) For each NF sound, identify the likely trigger: (a) physiological, (b) cognitive load, (c) anxiety.
(3) Re-record the same passage with a deliberate strategy to eliminate NF sounds. Compare NF counts across both recordings.
Target: reduce NF paralinguistic differentiator frequency in the second recording.

**Weekly Submission and Feedback Protocol**

Each week, participants submitted their recorded practice output and written reflection via the online platform. The research team provided individualized written feedback within 72 hours, addressing: (a) accuracy of paralinguistic feature identification or classification, (b) quality of prosodic/rate/pause/repair strategy, and (c) one specific improvement target for the following week. Feedback was standardized using a rubric to ensure consistency across participants and annotators.

**Table S5. Weekly submission feedback rubric.**

| **Dimension** | **Criteria** | **Score (0–3)** |
| --- | --- | --- |
| Feature identification accuracy | Correct classification of paralinguistic features per module criteria | 0 = <50%; 1 = 50–69%; 2 = 70–84%; 3 = ≥85% |
| Strategy application | Evidence of proactive planning in production output | 0 = No evidence; 1 = Partial; 2 = Consistent; 3 = Fully integrated |
| Metacognitive reflection | Quality of written self-evaluation and target-setting | 0 = Absent; 1 = Superficial; 2 = Adequate; 3 = Insightful |

**References (Supplementary)**

*Braver, T. S. (2012). The variable nature of cognitive control: a dual mechanisms framework. Trends in Cognitive Sciences, 16(2), 106–113.*

*Flavell, J. H. (1979). Metacognition and cognitive monitoring: A new area of cognitive–developmental inquiry. American Psychologist, 34(10), 906–911.*

*Kormos, J. (2006). Speech production and second language acquisition. Lawrence Erlbaum.*

*Tang, F. (2020). Repair strategies in consecutive interpreting: Comparing professional interpreters and student interpreters. The Interpreter and Translator Trainer, 14(4), 424–444.*

*Trager, G. L. (1958). Paralanguage: A first approximation. Studies in Linguistics, 13(1–2), 1–12.*
